# Dissecting Interactions of *Saccharomyces cerevisiae* and *Pichia kudriavzevii* to Shape Kiwifruit Wine Flavor

**DOI:** 10.3390/foods13244077

**Published:** 2024-12-17

**Authors:** Yi-Wen Wang, Yi-Fen Huang, Ya-Qi Guo, Li Sun, Zhi-Lin Jiang, Yuan-Ting Zhu, Rui-Qi Zeng, Qi Li, Chen Xiao, Yong Zuo

**Affiliations:** 1 Key Laboratory of the Evaluation and Monitoring of Southwest Land Resources (Ministry of Education), Sichuan Normal University, Chengdu 610101, China; 20221501047@stu.sicnu.edu.cn (Y.-W.W.); jzl13438550280@163.com (Z.-L.J.); 2 College of Life Science, Sichuan Normal University, Chengdu 610101, China; 20211501042@stu.sicnu.edu.cn (Y.-F.H.); 15883436538@163.com (Y.-Q.G.); sl4104@163.com (L.S.); zhuyuantingwode@163.com (Y.-T.Z.); 20230054@sicnu.edu.cn (R.-Q.Z.); xiaoqi@sicnu.edu.cn (Q.L.)

**Keywords:** kiwifruit wine, non-*Saccharomyces*, mixed culture fermentation, transcriptome analysis, differential gene expression

## Abstract

Mixed fermentation with *Saccharomyces cerevisiae* and *Pichia kudriavzevii* has been shown to enhance wine aroma, yet the underlying mechanisms remain unclear. Monoculture of *S. cerevisiae*, monoculture of *P. kudriavzevii*, and mixed culture of *S. cerevisiae* and *P. kudriavzevii* were conducted, and the study analyzed and compared the biomass, flavor profile, and transcriptome responses of the three groups. Both yeast species exhibited growth inhibition in mixed culture, especially *P. kudriavzevii*. Significant differences were observed in three organic acids and the foremost 20 volatile compounds. Mixed fermentation enhanced esters (e.g., ethyl butyrate, isoamyl acetate) and volatile acids (e.g., hexanoic acid), but decreased isobutanol, phenylethyl alcohol, and quinic acid. Transcriptomic analysis revealed 294 and 332 differentially expressed genes (DEGs) in *S. cerevisiae* and *P. kudriavzevii*, respectively. The Kyoto Encyclopedia of Genes and Genomes (KEGG) annotation results indicated that DEGs in mixed fermentation were concentrated in carbohydrate metabolism and amino acid metabolism. Our integrated analysis suggested that genes such as *TDH2*, *TDH3*, and *ENO2* were pivotal for ester biosynthesis. Moreover, *ADH1*, *ADH2*, *HPA3*, *ALD6*, and *ARO8* were associated with quinic acid synthesis. Furthermore, *ILV2*, *ILV5*, *ALD6*, and others were central to the production of isobutanol and phenylethyl alcohol.

## 1. Introduction

Kiwifruit, belonging to the genus *Actinidia* in the family Actinidiaceae, is not only highly tasty but also rich in dietary fiber, amino acids, polyphenols, vitamins, and minerals, earning it the title of “King of Vitamin C” [1,2]. Being a climacteric fruit, kiwifruit undergoes a rapid softening and decay process post-harvest, which presents significant challenges for long-term preservation [3]. Kiwifruit wines, with their abundant nutrients and low alcohol levels, have become increasingly popular as consumers show a growing preference for healthier drink options [4]. Therefore, processing kiwifruit into wine not only preserves its abundant nutritional content but also enhances its economic value [5]. Currently, the production of kiwifruit wine primarily relies on commercial wine yeasts, which exhibit high fermentation efficiency and alcohol tolerance but produce wines with a poor aroma profile, resulting in severe product homogeneity [5,6].

While *Saccharomyces cerevisiae* is the predominant yeast species in fruit wine fermentation, non-*Saccharomyces* yeasts (over 200 species) are also present in significant quantities [7,8]. These microorganisms were traditionally viewed as undesirable contaminants in winemaking due to their production of volatile acids [9]. Recently, an increasing number of studies have shown that non-*Saccharomyces* yeasts can reduce ethanol content, modulate the nutritional components of wine, improve wine stability and clarity, and alter the flavor profile and aroma of wine during fermentation [10,11,12,13]. Therefore, co-fermentation of non-*Saccharomyces* and *Saccharomyces* yeasts is a promising approach to enhance the quality of kiwifruit wine and develop new products.

Recent studies have shown that co-fermentation using non-*Saccharomyces* and *Saccharomyces* yeasts not only maintains fermentation efficiency but also promotes the formation of positive flavors such as floral, fruity, and nutty aromas [14,15,16]. The aroma of kiwifruit wine primarily originates from the complex metabolic activities of microorganisms during fermentation, which is influenced by the interactions between microorganisms and substrates, as well as among microorganisms themselves [17,18,19]. Although the interaction between non-*Saccharomyces* and *Saccharomyces* yeasts has been studied, the underlying mechanisms remain complex.

The co-fermentation of fruits using *P. kudriavzevii* (a non-*Saccharomyces* yeast) and *S. cerevisiae* has been studied to explore the effects on wine flavor and quality. For instance, several studies described that sequential and mixed culture of *S. cerevisiae* with *P. kudriavzevii* increased the concentrations of phenylethyl alcohol, higher alcohols, and ethyl esters, while decreasing fatty acids and C6-alcohols concentrations, which enhanced the fruity flavor, floral aroma, and sensory evaluation of wine [20,21,22]. However, few studies investigate the impact of the co-fermentation of *S. cerevisiae* and *P. kudriavzevii* on flavor compounds at gene expression level.

Therefore, in this study, monoculture and mixed culture fermentation of *S. cerevisiae* and *P. kudriavzevii* were conducted to investigate the effects on kiwifruit wine production. By comparing the physicochemical properties, flavor profiles, and transcriptional changes between different fermentations, we sought to clarify the synthetic metabolic pathways of key differential flavor compounds and elucidate the underlying mechanisms governing their interactions. This study could provide valuable information on the production of kiwifruit wine with a complex flavor profile and fermentation process optimization.

## 2. Materials and Methods

### 2.1. Kiwifruit Wine Production and Sample Collection

The kiwifruit used in the study was Xuxiang kiwifruit from Xi’an, Shaanxi Province. Approximately 1000 g of fresh kiwifruits with 8–9 maturity stages were selected. The pulp and water were blended in a 1:1 (*v*/*v*) ratio for 30 s using a juicer. Pectinase was added at a concentration of 0.45 g/L and incubated at 25 °C for 24 h. Potassium metabisulfite was then added at a concentration of 56 mg/L, and the soluble solid content (SSC) was adjusted to 21°Brix, while the pH was adjusted to 3.5. After centrifugation, the supernatant of the kiwi juice was filtered through a 0.22 μm membrane, and the sterility of the kiwi juice was verified by plate counting. Activated yeast was then inoculated into the kiwi juice to a cell density of 1 × 10^7^ CFU/mL.

Fermentation was carried out at a constant temperature of 28 °C for 7 days. Three biological replicates were set up, and samples were taken every 24 h. After fermentation, 1 mL of the fermentation broth was sampled to determine the yeast biomass. Then, 1 mL of fermentation broth from the total fermentation broth was centrifuged at 9000 rpm for 10 min, and the supernatant was collected for the determination of reducing sugar, alcohol content, organic acids, and volatile compounds, respectively. *S. cerevisiae*, derived from instant dry yeast (Angel Yeast Co., Ltd., Yichang, China), was selected for monoculture fermentation (referred to as group S). Additionally, a laboratory-screened strain of *P. kudriavzevii* was chosen as the non-*Saccharomyces* yeast for monoculture fermentation (referred to as group P). A mixed culture fermentation group (MIX) was prepared by mixing equal proportions of *S. cerevisiae* (MIX-S) and *P. kudriavzevii* (MIX-P).

### 2.2. Yeast Biomass Determination and Physicochemical Analysis

Samples were taken every 24 h from 0 h post-inoculation until the end of fermentation. The fermentation samples were serially diluted with sterile water and spread onto WL differential medium, the preparation of which was described by Li et al. [23]. After incubation at 28 °C for 2 days, the colonies on the plates were counted to determine the viable cell counts, and the final results were expressed as colony-forming units per mL.

*S. cerevisiae* and *P. kudriavzevii* differed morphologically on WL differential medium, with the specific differences shown in Supplementary Figure S1. Thus, the two strains used in this study could be distinguished on WL differential medium and viable cells counted.

The pH and the soluble solid content of kiwifruit juice and kiwifruit wine were measured using a pH meter (ST2100; Ohaus Instrument Co., Ltd., Changzhou, China) and a hand-held refractometer (WYT-J; Shanghai HuYueMing Scientific Instrument Co., Ltd., Shanghai, China). The ethanol and reducing sugar content of kiwifruit wines were determined with reference to the spectrophotometry method [24] and 3,5-dinitrosalicylic acid colorimetric analysis [25] reported in previous studies, respectively.

### 2.3. Organic Acids and Volatile Compounds Analysis

Organic acids were determined using the LC-2030 system (Shimadzu, Kyoto, Japan). The sample entered the Agilent ZORBAX SB-Aq column (4.6 mm internal diameter × 250 mm length; 5 μm particle size), and the mobile phase was 0.04 mol/L KH_2_PO_4_-Methanol solution (*v*/*v* = 99:1, pH = 2.7). The wavelength, column temperature, injection volume, and flow rate were 210 nm, 30 °C, 20 μL, and 0.8 mL/min, respectively. Mixed standard solutions of different concentrations of organic acids (oxalic acid, quinic acid, malic acid, lactic acid, citric acid, and fumaric acid) were configured, and the concentration of organic acids in kiwifruit juice and kiwifruit wine was determined using the external standard method.

With reference to a previous study [5,26], the volatile compounds were extracted and analyzed using headspace solid-phase microextraction coupled with gas chromatography–mass spectrometry (HS-SPME-GC-MS). Each sample (1 mL) was mixed with 4 mL saturated NaCl solution and 10 μL 2-octanol (0.0822 mg/mL, as internal standard) in a headspace vial. A well-aged 50/30 μm DVB/CAR/PDMS fiber (Supelco, Bellefonte, PA, USA) extracted volatiles at 60 °C for 45 min. GC-MS (Shimadzu QP2020, Shimadzu, Japan) analysis was performed on an HP-INNOWax column (60 m × 0.25 mm i.d., 0.25 μm film thickness; J&W Scientific, Folsom, CA, USA) with helium as the carrier gas (1.0 mL/min). The temperature increase program was as follows: first at 40 °C for 5 min, followed by an increase to 100 °C at a rate of 5 °C/min, then an increase to 230 °C at a rate of 6 °C/min, and held for 10 min. The electron impact (EI) mode was set at 70 eV, with a scanning mass-to-charge (*m*/*z*) range from 40 to 450, and the interface and ionization source temperatures were 250 °C and 230 °C, respectively.

The compounds were identified by comparison with mass spectra data of the NIST-14 mass spectral database (similarity > 80%). Semi-quantitation of the volatile compounds was calculated using 2-octanol as an internal standard and applying the formula *C*_2_ = 10^4^ × *C*_1_ × *A*_2_/*A*_1_, where *C*_2_ is the relative concentration of the volatile compound (μg/L kiwifruit wine), *C*_1_ is the concentration of the internal standard (0.0822 mg/mL), *A*_2_ is the peak area of one volatile compound in the sample, and *A*_1_ is the peak area of the internal standard in the sample [26].

### 2.4. Transcriptome Analysis

The RNA-sequencing was carried out for three biological replicates of different fermentation groups (S, P, and MIX). Yeast cells were separated from the fermentation samples by centrifugation (10,000 rpm, 4 °C for 2 min) and immediately frozen in liquid nitrogen. The total RNA was extracted using TRIzol^®^ Reagent according to the manufacturer’s instructions. The concentration of the total RNA was measured by NanoDrop^®^ 2000 (Thermo Scientific, Waltham, MA, USA), and the quality was determined by 5300 Bioanalyser (Agilent Technologies, Santa Clara, CA, USA). High-quality RNA samples (OD260/280 ≥ 1.8, OD260/230 ≥ 1.0) were submitted to Majorbio Biotech Co., Ltd. (Shanghai, China) for cDNA library construction and sequencing.

After sequencing, the trimming and quality control of raw reads were performed using fastp v0.19.5 (https://github.com/OpenGene/fastp, accessed on 25 July 2024) with the default settings. For RNA-seq, clean reads were mapped to the reference genome of *S. cerevisiae* S288C (https://www.ncbi.nlm.nih.gov/datasets/genome/?taxon=559292, accessed on 25 July 2024) and *P. kudriavzevii* ASM305444v1 (https://www.ncbi.nlm.nih.gov/datasets/genome/GCF_003054445.1/, accessed on 25 July 2024) using the Hisat2 v2.1.0 [27]. To identify DEGs between two different samples, the expression level of each transcript was calculated according to the transcripts per million (TPM) reads method. RSEM v1.3.3 [28] was used to quantify gene abundances. Essentially, differential expression analysis was performed using the DESeq2 v1.24.0 [29]. DEGs with |log_2_ FC| ≥ 1 and FDR < 0.05 (DESeq2) were considered to be significantly different expressed genes. In addition, a functional enrichment analysis was applied to the full set of DEGs, based on the Gene Ontology (GO) and the Kyoto Encyclopedia of Genes and Genomes (KEGG) databases, taking advantage of the software tools Goatools v 0.6.5 (https://github.com/tanghaibao/Goatools, accessed on 28 July 2024) and Python scipy v1.8.0 software, respectively.

### 2.5. Statistical Analysis

Data analysis was performed using one-way analysis of variance (ANOVA) by SPSS Statistics v25.0 (SPSS, Inc., Chicago, IL, USA). The differential volatile compounds were investigated by applying the partial least squares-discriminant analysis (PLS-DA) by SIMCA v14.1 (Sartorius Stedim, Malmö, Sweden). The significant differences among the data were determined using the Duncan test, at *p* < 0.05. The results were expressed as mean ± standard deviation (SD).

## 3. Results and Discussion

### 3.1. The Growth Dynamics of Yeasts During Fermentation

**Figure 1:**
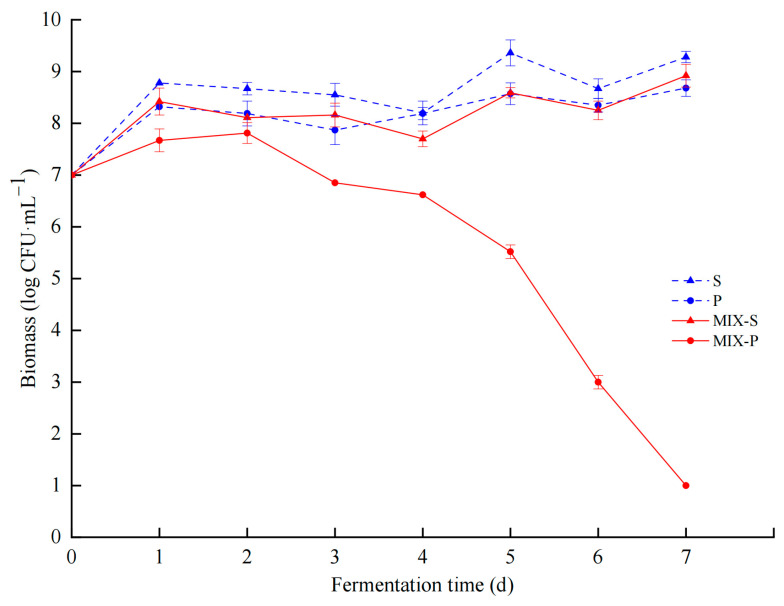
Growth dynamics of yeasts during fermentation process. Note: *S. cerevisiae* in monoculture fermentation (S), *P. kudriavzevii* in monoculture fermentation (P), *S. cerevisiae* in mixed culture fermentation (MIX-S), and *P. kudriavzevii* in mixed culture fermentation (MIX-P).

The biomass of different fermentation groups in this study was compared (Figure 1). During the monoculture fermentation, the biomass of *S. cerevisiae* (S) and *P. kudriavzevii* (P) exhibited similar growth patterns. On the first day of the fermentation process, the cell counts of both strains increased rapidly from 7 log CFU/mL to 8.65 ± 0.01 log CFU/mL (S) and 8.17 ± 0.04 log CFU/mL (P), respectively, and then remained relatively stable at 8–9 log CFU/mL until the end of fermentation.

In the mixed fermentation group (MIX), the biomass of *S. cerevisiae* (MIX-S) showed a similar trend to that in the monoculture fermentation, fluctuating between 7.72 ± 0.04 log CFU/mL and 8.93 ± 0.03 log CFU/mL throughout the fermentation process. However, the cell count was lower than that in the monoculture, suggesting that the growth of *S. cerevisiae* (MIX-S) was inhibited by *P. kudriavzevii* (P) during mixed culture fermentation. Compared to the growth of *S. cerevisiae*, the biomass of *P. kudriavzevii* (MIX-P) was more significantly affected. Its biomass reached a peak of 7.78 ± 0.03 log CFU/mL on the 2nd day of fermentation and then decreased rapidly, with a final cell count of 0.97 ± 0.09 log CFU/mL at the end of the fermentation. This indicated that the growth of *P. kudriavzevii* was inhibited significantly by *S. cerevisiae* during mixed culture fermentation.

This phenomenon had also been reported [17,30]; it was speculated that nutrient competition and the cell–cell contact-mediated mechanism are the primary factors driving the inhibition of non-*Saccharomyces* yeast growth during co-fermentation.

### 3.2. Dynamics of Reducing Sugar and Alcohol Content During Fermentation

**Figure 2:**
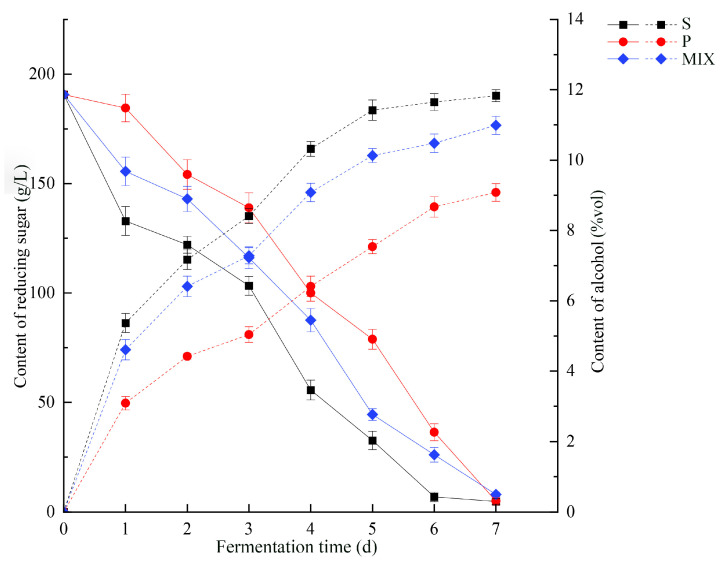
Dynamics of alcohol content and reducing sugars during fermentation process. Note: In the graph, the solid line represents the change in content of reducing sugar concentration, and the dashed line represents the change in content of ethanol.

The changes in the reducing sugar and alcohol content during both the monoculture and mixed culture fermentation can be seen in Figure 2. After the 7th day of fermentation, reducing sugar in both the monoculture and mixed culture groups was fully utilized in the fermentation system, showing a decreasing trend among different groups.

In the early stage of fermentation, the reducing sugar consumption rate of the *S. cerevisiae* monoculture group (S) and the mixed culture group (MIX) was higher than that of the *P. kudriavzevii* monoculture group (P). At the end of fermentation, the residual sugar content in the three groups ranged from 4.5 ± 0.17 to 8.0 ± 0.09 g/L. Both the monoculture groups (S, P) and the mixed culture group (MIX) started producing ethanol from the first day of fermentation until the end. Among them, the monoculture group (S) produced ethanol at the fastest rate and with the highest content, followed by the mixed culture group (MIX), while the *P. kudriavzevii* monoculture group (P) had the lowest ethanol production rate and content. At the end of fermentation, the alcohol content of *S. cerevisiae* reached 11.86 ± 0.17%vol in the monoculture (S), 10.99 ± 0.26%vol in the mixed culture (MIX), and 9.08 ± 0.25%vol in the monoculture (P). Combined with the changes in yeast biomass, the biomass of *S. cerevisiae* (MIX-S) in the mixed culture group was not significantly affected by co-culture, which might be the reason why the ethanol content in the mixed culture could be maintained at a higher level.

### 3.3. Analysis of DEGs During Fermentation

To better investigate the interactions between the two yeasts, we employed transcriptomic analysis to study their differential gene expression. Based on the changes in the biomass of yeasts, content of ethanol and reducing sugar during fermentation, we selected the 3rd day of fermentation as the time point to investigate the interactions between *P. kudriavzevii* and *S. cerevisiae*.

**Figure 3:**
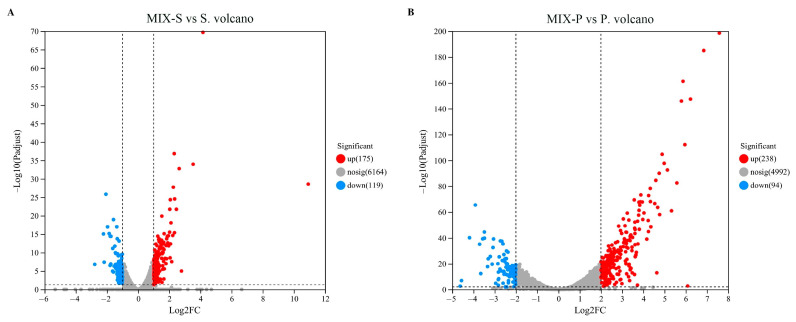
Volcano map of differential genes expression. (**A**) group MIX-S vs. group S, (**B**) group MIX-P vs. group P.

After quality control of the raw sequencing data, the DEGs of the mixed fermentation group (MIX) was compared with the monoculture fermentation groups (S and P), respectively (Figure 3); a total of 294 DEGs were identified in MIX-S on the third day of fermentation, including 175 upregulated genes and 119 downregulated genes. In MIX-P, a total of 332 DEGs were identified on the third day of fermentation, including 238 upregulated genes and 94 downregulated genes. DEGs (|log2 FC| ≥ 1) were selected for further analysis (Supplementary Tables S1 and S2).

### 3.4. Functional Annotation Analysis of DEGs

To elucidate the functional implications of the identified DEGs, Gene Ontology (GO) annotation analysis was performed. The GO terms were categorized into three main ontologies: cellular component (CC), biological process (BP), and molecular function (MF).

In the MIX-S group, DEGs were distributed as 8 in BP, 7 in CC, and 5 in MF and significantly enriched in biological processes related to cellular processes and metabolism. Cellular components such as cell part and organelle were overrepresented, while molecular functions primarily involved binding and catalytic activities. Similarly, in the MIX-P group, DEGs were distributed as 6 in BP, 7 in CC, and 7 in MF and predominantly associated with cellular processes and metabolism. Cellular components, including cell part and membrane, were significantly enriched. Moreover, DEGs were enriched for proteins involved in binding and catalytic activities (Supplementary Figure S2).

To further understand the functions of DEGs, we concurrently performed the Kyoto Encyclopedia of Genes and Genomes (KEGG) database annotation. All DEGs in MIX-S and MIX-P were annotated against KEGG using S and P as reference groups, respectively. In MIX-S, 201 DEGs were categorized into 17 level-2 KEGG pathways, which fell into four broad functional categories: metabolism, genetic information processing, environmental information processing, and cellular processes.

**Figure 4:**
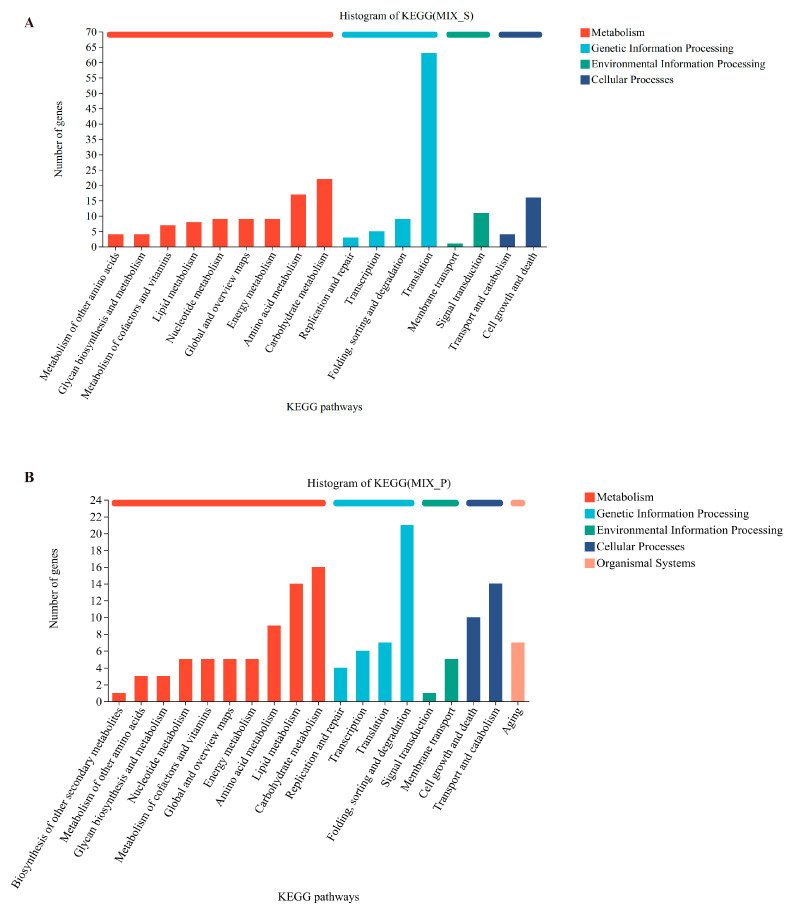
KEGG analysis of differential genes. (**A**) group MIX-S vs. group S, (**B**) group MIX-P vs. group P.

The most significantly enriched pathway in MIX-S was translation (63 DEGs), encompassing processes such as ribosome biogenesis (map03008), nucleocytoplasmic transport (map03013), and overall ribosome function (map03010). Carbohydrate metabolism (22 DEGs) was the second most enriched pathway, with key sub-pathways including the citric acid cycle (TCA cycle, map00020), starch and sucrose metabolism (map00500), and glycolysis/gluconeogenesis (map00010) (Figure 4A).

In MIX-P, 141 DEGs were categorized into 19 level-2 KEGG pathways spanning five functional categories. The most enriched pathway was protein folding, sorting, and degradation (21 DEGs), with key sub-pathways including protein export (map03060) and ubiquitin-mediated proteolysis (map04120). Similar to MIX-S, carbohydrate metabolism (16 DEGs) was also enriched, with sub-pathways such as the pentose phosphate pathway (map00040) and the TCA cycle (map00020) being represented (Figure 4B).

### 3.5. Analysis of Genes Involved in the Biosynthesis Pathways of Certain Differential Flavor Compounds

**Figure 5:**
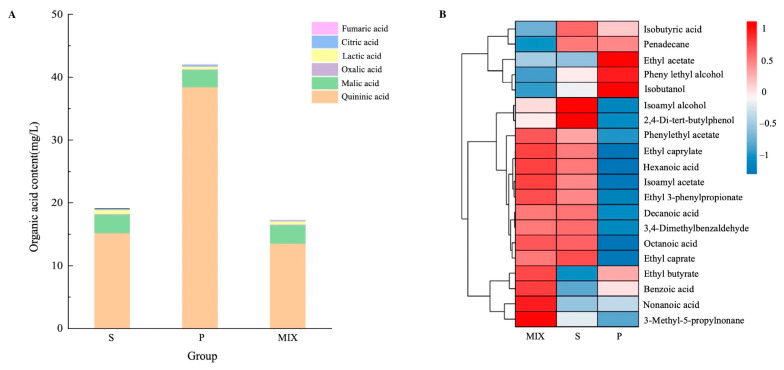
Differential metabolic profiles among various fermentation groups. (**A**) organic acids, (**B**) volatile compounds.

To further elucidate the impact of mixed fermentation on flavor compounds, the potential biosynthetic pathways were investigated by combining flavor compounds and transcriptomic data. The organic acid profiles of samples from different groups were compared (Figure 5A). The monoculture fermentation of *P. kudriavzevii* exhibited the highest total organic acid content of 42.04 ± 2.20 mg/mL, with quinic acid, lactic acid, and malic acid being the predominant organic acids. The mixed fermentation group (MIX) had the lowest quinic acid content of 113.52 ± 1.70 mg/mL, while the levels of lactic acid (0.54 ± 0.11 mg/mL) and malic acid (2.90 ± 0.39 mg/mL) in the MIX group were not significantly different from those in the other monoculture fermentation groups.

A heatmap of the foremost 20 most differentiated volatile compounds identified by PLS-DA analysis among the groups is shown in Figure 5B, Supplementary Table S3. The major differential volatile compounds in the ester category were ethyl acetate, ethyl butyrate, and isoamyl acetate, etc.; in the alcohol and acid categories, they were isobutanol, phenylethyl alcohol, hexanoic acid, octanoic acid, and nonanoic acid, etc. Additionally, other flavor compounds, such as 2,4-di-tert-butylphenol and 3-methyl-5-propylnonane, were identified. Compared to the monoculture fermentation groups of *S. cerevisiae* (S) and *P. kudriavzevii* (P), the mixed fermentation group (MIX) had lower contents of isobutanol (70.70 ± 6.95 μg/L), isoamyl alcohol (1018.75 ± 45.44 μg/L), and phenylethyl alcohol (687.88 ± 24.42 μg/L). The MIX group exhibited significantly elevated levels of ethyl butyrate, isoamyl acetate, ethyl caprylate, phenylethyl acetate, and ethyl 3-phenylpropionate compared to the monoculture fermentation group of *S. cerevisiae* (S), and this result was similar to the findings reported by Sun et al. [5]. These esters imparted fruity, floral, and sweet flavors to kiwifruit wines [16,31]. Moreover, the MIX group produced higher quantities of all six ester compounds except for ethyl acetate when compared to the monoculture fermentation group of *P. kudriavzevii* (P). Additionally, the MIX group demonstrated superior production of hexanoic acid (145.33 ± 10.33 μg/L), octanoic acid (981.1 ± 106.34 μg/L) and nonanoic acid (35.17 ± 0.41 μg/L) in comparison to both monoculture groups S and P. The presence of fatty acids could increase the complexity of wine aroma [20,31]. A higher content could impart negative sensory attributes like rancid; however, at appropriate concentrations, they might effectively prevent ester hydrolysis and contribute to a balance in the wine’s aroma [20].

Building upon previous findings [31,32,33,34], we employed an integrative approach to analyze differential gene expression and volatile profiles, aiming to elucidate the underlying mechanisms associated with key differential metabolites and provide insights into the complex metabolic pathways involved.

#### 3.5.1. Expression Analysis of DEGs Involved in Alcohol Biosynthesis

**Figure 6:**
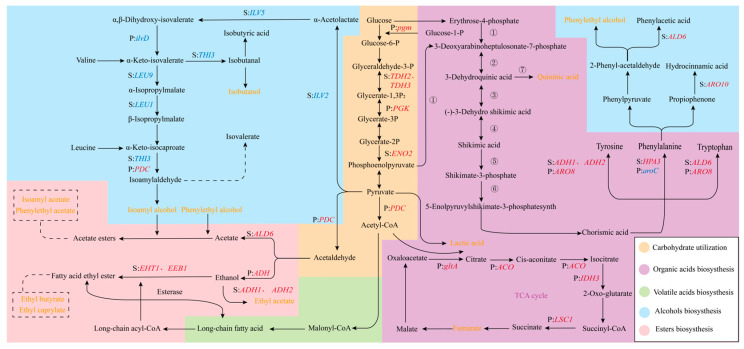
Metabolic pathways of alcohol, ester, non-volatile acid, and volatile acid production by yeast during fermentation. Note: The colors of the regions represent different flavor formation or substrate utilization pathways. Italicized genes encode enzymes involved in the metabolic pathways; red indicates upregulation; blue indicates downregulation; and orange indicates characteristic flavor compounds. ① 3-deoxy-d-arabinoheptulosonate-7-phosphate synthase; ② 3-dehydroquinic acid synthase; ③ 3-dehydroquinic acid dehydratase; ④ shikimate dehydrogenase; ⑤ shikimate kinase; ⑥ 5-enolpyruvyl shikimate-3-phosphate synthase; ⑦ dehydroquinate dehydrogenase.

The content of alcohols significantly influences the aroma and quality of alcoholic beverages, serving as a crucial indicator for assessing beverage quality [35]. Higher alcohols can be synthesized through two primary pathways: the Ehrlich pathway [36] and the sugar metabolism pathway (as illustrated in Figure 6). Although multiple genes encode enzymes involved in the Ehrlich pathway, our understanding of these enzymes remains limited [31]. In this study, three higher alcohols with significant differences in content were identified: isoamyl alcohol, isobutanol, and phenylethyl alcohol. In the Ehrlich pathway, these three alcohols can be produced from leucine, valine, and phenylalanine, respectively [37,38].

Compared to the monoculture fermentation group S, the mixed fermentation group (MIX) exhibited a reducing trend in isobutanol, isoamyl alcohol, and phenylethyl alcohol. The decrease in isobutanol and isoamyl alcohol levels may be attributed to the downregulation of genes involved in the Ehrlich and Harris pathways, including *ILV2* (Log_2_ FC = −0.19), *ILV5* (Log_2_ FC = −0.09), *LEU1* (Log_2_ FC = −0.18), *LEU9* (Log_2_ FC = −0.44), and *THI3* (Log_2_ FC = −0.28) [39,40]. Although the downregulation of these genes was not statistically significant, their combined effects on these pathways could have led to a decrease in isobutanol and isopentanol production. The decrease in phenylethyl alcohol content might be associated with the upregulation of *ARO10* (Log_2_ FC = 0.09) and *ALD6* (Log_2_ FC = 1.25, *p* < 0.05), genes involved in the synthesis of hydrocinnamic acid and phenylacetic acids. The increased consumption of phenylalanine for the production of these acids likely led to a decrease in phenylethyl alcohol production.

Compared to the monoculture fermentation group P, the MIX group showed decreased contents of isobutanol and phenylethyl alcohol. The decrease in isobutanol content may be attributed to the downregulation of *ilvD* (Log_2_ FC = −1.15), a key gene encoding an enzyme involved in the Harris pathway [40], while the decrease in phenylethyl alcohol was due to the downregulation of the gene *aroC* (Log_2_ FC= −1.56) in MIX-P encoding phenylalanine synthesis. The increase in isopentanol content could be attributed to the upregulation of *PDC* (Log_2_ FC = 2.93, *p* < 0.05), a key gene involved in leucine degradation in MIX-P, indicating that the increased consumption of leucine promoted the accumulation of isoamyl alcohol [38].

#### 3.5.2. Expression Analysis of DEGs Involved in Esters Biosynthesis

Esters produced by yeast metabolism contribute significantly to the floral and fruity aromas of fruit wines [41]. Ester synthesis primarily involves three pathways: the formation of acetate esters from higher alcohols and coenzyme A; the synthesis of acetate esters from ethanol and coenzyme A; and the formation of ester compounds from medium-chain fatty acids, coenzyme A, and ethanol [34] (Figure 6). Six esters were identified with significant differences: ethyl acetate, ethyl butyrate, isoamyl acetate, ethyl caprylate, ethyl caprate, phenylethyl acetate, and ethyl 3-phenylpropionate (Figure 5).

Compared to the monoculture group S, the MIX group exhibited higher levels of six differential esters. Isoamyl acetate, phenylethyl acetate, and ethyl acetate were likely synthesized from alcohols and acetyl-CoA. The increased contents of these esters were associated with the upregulation of key genes in the glycolytic pathway of MIX-S, including *TDH2* (Log_2_ FC = 2.46, *p* < 0.05), *TDH3* (Log_2_ FC = 2.08, *p* < 0.05), encoding glyceraldehyde-3-phosphate dehydrogenase, and *ENO2* (Log_2_ FC = 2.78, *p* < 0.05), encoding phosphoenolpyruvate hydratase. The upregulation of these three key enzymes promoted the production of pyruvate through glycolysis of MIX-S, which might further convert to acetaldehyde. Acetaldehyde was then oxidized to acetate by acetaldehyde dehydrogenase encoded by the *ALD6* gene [42] (Log_2_ FC = 1.25, *p* < 0.05), followed by the formation of corresponding acetate esters (isoamyl acetate and phenylethyl acetate). Alternatively, acetaldehyde could be converted to ethanol and then to ethyl acetate by acetaldehyde dehydrogenases encoded by the *ADH1* (Log_2_ FC = 1.73, *p* < 0.05) and *ADH2* (Log_2_ FC = 1.38, *p* < 0.05) genes. Ethyl butyrate and ethyl caprylate were synthesized from medium-chain fatty acyl-CoA and ethanol. The upregulation of key genes *EHT1* (Log_2_ FC = 0.20) and *EEB1* (Log_2_ FC = 0.01), which encode alcohol acyltransferases, might have led to an increase in its content.

The increased contents of six ester compounds were associated with the upregulation of key genes involved in glycolysis, specifically *PDC* (Log_2_ FC = 2.93, *p* < 0.05) and *pgm* (Log_2_ FC = 2.00, *p* < 0.05), as well as two isoforms of alcohol dehydrogenase (*ADH*; gene-C5L36_0D04320, Log_2_ FC = 4.03, *p* < 0.05 and gene-C5L36_0D04330, Log_2_ FC = 3.95, *p* < 0.05). The observed decrease in ethyl acetate content could be attributed to the substantial consumption of acetyl-CoA, which was a precursor for fatty acid ester biosynthesis.

#### 3.5.3. Expression Analysis of DEGs Involved in Acids Biosynthesis

Acids play a pivotal role in winemaking, exerting a significant influence on the quality, flavor, taste, and stability of wine. In volatile acids, the MIX group exhibited higher levels of hexanoic acid, octanoic acid, and nonanoic acid compared to the monoculture groups (S and P) [43]. Esterases have a bi-directional effect on the synthesis and hydrolysis of esters. They act on medium-chain fatty acid esters to catalyze the breaking of ester bonds, resulting in the breakdown of medium-chain fatty acid esters into fatty acids and alcohols. The metabolomics results showed that the content of medium-chain fatty acid esters in the MIX group was significantly higher than that in the S and P groups, which might be catalyzed by esterases to decompose to form fatty acids when they accumulated to a certain level (Figure 6).

In organic acids, quinic acid was a major acid [18] in kiwifruit wine and its biosynthesis was associated with the shikimate pathway (Figure 6): glucose can be converted to phosphoenolpyruvate (PEP) and erythrose 4-phosphate (E4P) through the embden-meyerhof pathway (EMP) and hexose monophosphate pathway (HMP), respectively. These two products entered the shikimate pathway under the catalysis of 3-Deoxy-d-arabino-heptulosonate-7-phosphate (DAHP) synthase [44], eventually leading to the synthesis of tyrosine, tryptophan, and phenylalanine [45]. Quinic acid was a by-product of the shikimate pathway. When shikimate accumulated to a certain concentration, it was secreted out of the cell and then transported back into the cytoplasm to generate quinic acid in the reverse direction of shikimate synthesis. The quinic acid content in group S was 15.16 ± 3.60 mg/mL, and 18.62 ± 2.97 mg/mL in group P. Compared to monoculture groups S and P, the quinic acid content in the MIX group decreased to 13.52 ± 1.70 mg/mL.

Comparing the MIX group to group S, the decrease in quinic acid content was associated with the upregulation of genes encoding enzymes involved in tyrosine metabolism (*ADH1*, Log_2_ FC = 1.73, *p* < 0.05; *ADH2*, Log_2_ FC = 1.38, *p* < 0.05), phenylalanine metabolism (*HPA3*, Log_2_ FC = 1.00, *p* < 0.05), and tryptophan metabolism (*ALD6*, Log_2_ FC = 1.25, *p* < 0.05) in MIX-S. The upregulation of these genes promoted the conversion of shikimate to corresponding amino acids through the branched-chain amino acid pathway, thereby preventing the accumulation of shikimate and ultimately reducing the quinic acid production. When comparing the MIX group to the group P, the decrease in quinic acid content was due to the upregulation of the gene *ARO8* (Log_2_ FC = 1.24), which is involved in the metabolism of tyrosine, and tryptophan in MIX-P.

The increased lactate content could be a consequence of upregulated gene *PGK* (Phosphoglycerate Kinase, Log_2_ FC = 1.17), a key enzyme in glycolysis. This upregulation stimulated glycolysis, leading to elevated pyruvate levels, which were subsequently converted to lactate by lactate dehydrogenase. In contrast, the elevated fumarate content was associated with the upregulation of multiple genes, including *gltA* (Log_2_ FC = 2.60, *p* < 0.05), *ACO* (Log_2_ FC = 1.38), *IDH3* (Log_2_ FC = 1.31), and *LSC1* (Log_2_ FC = 1.62), which are essential for the TCA cycle. The enhanced expression of these genes promoted TCA cycle activity, resulting in increased fumarate production.

## 4. Conclusions

This study revealed the interactions between *S. cerevisiae* and *P. kudriavzevii* during mixed fermentation and their impact on metabolic products, highlighting the significance of these interactions for optimizing fermentation processes and improving product quality. Changes in biomass, physicochemical properties, and flavor compounds during the different fermentations were monitored. Biomass measurements and metabolic results indicated that *P. kudriavzevii* in mixed culture fermentation (MIX-P) produced less biomass, and after 7 days it decreased almost to 0 (Figure 1). Monoculture P produced less alcohol and reduced sugar decreased slowly; however, all mixtures after 7 days reached value 0 (Figure 2). Based on the observed changes in yeast biomass, ethanol content, and reducing sugar levels during fermentation, the 3rd day was selected as the optimal time point to investigate the interactions between *P. kudriavzevii* and *S. cerevisiae*. Transcriptomic analysis revealed 294 and 332 differentially expressed genes (DEGs) in *S. cerevisiae* and *P. kudriavzevii*, respectively (Figure 3). These DEGs in MIX-S were categorized into the four pathway categories of the Kyoto Encyclopedia of Genes and Genomes (KEGG) – metabolism, genetic information processing, environmental information processing, and cellular processes –, whereas the DEGs in MIX-P were categorized into the five pathway categories of the KEGG, with one more organismal systems (Figure 4). The monoculture fermentation of *P. kudriavzevii* exhibited the highest total organic acid content, with quinic acid, lactic acid, and malic acid being the predominant organic acids. The mixed fermentation group (MIX) had the lowest quinic acid content, while contents of lactic acid and malic acid in the MIX group were not significantly different from those in the other monoculture fermentation groups (Figure 5A). The MIX group exhibited significantly elevated levels of most esters (ethyl butyrate, isoamyl acetate, ethyl caprylate, phenylethyl acetate, and ethyl 3-phenylpropionate), and volatile acids (hexanoic acid, octanoic acid, and nonanoic acid) compared to the monoculture fermentation groups (Figure 5B). By integrating transcriptomic and metabolomic data, key genes involved in the formation of differential flavor compounds were identified. Genes such as *TDH2*, *TDH3*, and *ENO2* were implicated in ester biosynthesis, while *ADH1*, *ADH2*, *HPA3*, *ALD6*, and *ARO8* were associated with quinic acid synthesis. Furthermore, *ILV2*, *ILV5*, *ALD6*, and others were central to the production of isobutanol and phenylethyl alcohol (Figure 6). These findings provide valuable insights into the mechanisms underlying flavor compound formation in winemaking.

## Data Availability

The original contributions presented in this study are included in the article/Supplementary Materials; further inquiries can be directed to the corresponding authors.

## Supplementary Materials

The following supporting information can be downloaded at: https://www.mdpi.com/article/10.3390/foods13244077/s1, Figure S1: Colony morphologies of different yeast strains on WL identification medium; Figure S2: GO analysis of differential genes. (A) group MIX-S vs. group S, (B) group MIX-P vs group P; Table S1: Genes with significant expression differences between S vs. MIX-S; Table S2: Genes with significant expression differences between MIX-P vs. P; and Table S3: The volatile compounds of different fermentation groups (MIX, S, P).

## References

[1] Liang J., Ren Y., Wang Y., Han M., Yue T., Wang Z., Gao Z. (2021). Physicochemical, nutritional, and bioactive properties of pulp and peel from 15 kiwifruit cultivars. Food Biosci..

[2] Zhao Y., Wang P., Zhan P., Tian H., Lu C., Tian P. (2021). Aroma characteristics of cloudy kiwifruit juices treated with high hydrostatic pressure and representative thermal processes. Food Res. Int..

[3] Chai J., Liao B., Li R., Liu Z. (2022). Changes in taste and volatile compounds and ethylene production determined the eating window of ‘Xuxiang’ and ‘Cuixiang’ kiwifruit cultivars. Postharvest Biol. Technol..

[4] Zhou B.D., Liu X.C., Lan Q.Y., Wan F., Yang Z.B., Nie X., Cai Z.J., Hu B., Tang J.N., Zhu C.L. (2024). Comparison of aroma and taste profiles of kiwi wine fermented with/without peel by combining intelligent sensory, gas chromatography-mass spectrometry, and proton nuclear magnetic resonance. Foods.

[5] Sun W., Chen X., Feng S., Bi P., Han J., Li S., Guo J. (2024). Effect of sequential fermentation with indigenous non-*Saccharomyces cerevisiae* combinations and *Saccharomyces cerevisiae* on the chemical composition and aroma compounds evolution of kiwifruit wine. Food Chem..

[6] Liu S., Yang L., Zhou Y., He S., Li J., Sun H., Xu S. (2019). Effect of mixed moulds starters on volatile flavor compounds in rice wine. LWT.

[7] Jolly N.P., Varela C., Pretorius I.S. (2014). Not your ordinary yeast: Non-*Saccharomyces* yeasts in wine production uncovered. FEMS Yeast Res..

[8] Drumonde-Neves J., Fernandes T., Lima T., Pais C., Franco-Duarte R. (2021). Learning from 80 years of studies: A comprehensive catalogue of non-*Saccharomyces* yeasts associated with viticulture and winemaking. FEMS Yeast Res..

[9] Padilla B., Gil J.V., Manzanares P. (2016). Past and future of non-*Saccharomyces* yeasts: From spoilage microorganisms to biotechnological tools for improving wine aroma complexity. Front. Microbiol..

[10] Borren E., Tian B. (2020). The important contribution of Non-*Saccharomyces* yeasts to the aroma complexity of wine: A Review. Foods.

[11] Contreras A., Hidalgo C., Schmidt S., Henschke P.A., Curtin C., Varela C. (2015). The application of non-*Saccharomyces* yeast in fermentations with limited aeration as a strategy for the production of wine with reduced alcohol content. Int. J. Food Microbiol..

[12] Lin M.M.H., Boss P.K., Walker M.E., Sumby K.M., Grbin P.R., Jiranek V. (2020). Evaluation of indigenous non-*Saccharomyces* yeasts isolated from a South Australian vineyard for their potential as wine starter cultures. Int. J. Food Microbiol..

[13] Wei J., Zhang Y., Qiu Y., Guo H., Ju H., Wang Y., Yue T. (2020). Chemical composition, sensorial properties, and aroma-active compounds of ciders fermented with *Hanseniaspora osmophila* and *Torulaspora quercuum* in co- and sequential fermentations. Food Chem..

[14] Escribano-Viana R., González-Arenzana L., Portu J., Garijo P., López-Alfaro I., López R., Gutiérrez A.R. (2018). Wine aroma evolution throughout alcoholic fermentation sequentially inoculated with non-*Saccharomyces/Saccharomyces* yeasts. Food Res. Int..

[15] Rêgo E.S.B., Rosa C.A., Freire A.L., Machado A.M.d.R., Gomes F.d.C.O., Costa A.S.P.d., Padilha F.F. (2020). Cashew wine and volatile compounds produced during fermentation by non-*Saccharomyces* and *Saccharomyces* yeast. LWT.

[16] Yang X., Zhao F., Yang L., Li J., Zhu X. (2022). Enhancement of the aroma in low-alcohol apple-blended pear wine mixed fermented with *Saccharomyces cerevisiae* and non-*Saccharomyces* yeasts. LWT.

[17] Hu K., Zhao H., Edwards N., Peyer L., Tao Y., Arneborg N. (2022). The effects of cell-cell contact between *Pichia kluyveri* and *Saccharomyces cerevisiae* on amino acids and volatiles in mixed culture alcoholic fermentations. Food Microbiol..

[18] Huang J., Wang Y., Ren Y., Wang X., Li H., Liu Z., Gao Z. (2022). Effect of inoculation method on the quality and nutritional characteristics of low-alcohol kiwi wine. LWT.

[19] Zhang B., Zhang C., Li J., Zhou P., Lan Y., Duan C., Yan G. (2024). A comparative study to investigate the individual contribution of metabolic and physical interaction on volatiles formation in the mixed fermentation of *Torulaspora delbrueckii* and *Saccharomyces cerevisiae*. Food Microbiol..

[20] Liu W., Ji R., Aimaier A., Sun J., Pu X., Shi X., Wang B. (2023). Adjustment of impact phenolic compounds, antioxidant activity and aroma profile in Cabernet Sauvignon wine by mixed fermentation of *Pichia kudriavzevii* and *Saccharomyces cerevisiae*. Food Chem..

[21] Vicente J., Calderón F., Santos A., Marquina D., Benito S. (2021). High Potential of *Pichia kluyveri* and Other *Pichia* Species in Wine Technology. Int. J. Mol. Sci..

[22] Shi W.K., Wang J., Chen F.S., Zhang X.Y. (2019). Effect of *Issatchenkia terricola* and *Pichia kudriavzevii* on wine flavor and quality through simultaneous and sequential co-fermentation with *Saccharomyces cerevisiae*. LWT.

[23] Li C.Y., Zheng M.X., Zou Y.T., He Z.H., Xu B.T., Zhang Q., Qin J., Tian W.Q. (2024). Characteristics of Wild Cherry Beverage Co-fermented by *Hanseniaspora uvarum* and *Saccharomyces cerevisiae*. Sci. Technol. Food Ind..

[24] Wei X.X. (2016). Determination of Content of Alcohol in Wines by Colorimetry. Food Chem..

[25] Liu C.H., Zeng J.T., Bao Z.J., Li X., Zhu Z.J., Wang Z.S., Huang X.W. (2022). Determination of soluble sugar content in Mangifera indica Linn. by 3,5-dinitrosalicylic acid colorimetry. J. Food Saf. Qual..

[26] Xiao C., Wang L., Zhang Y.G., Tu T.Y., Wang S.T., Shen C.H., Zhong X.Z. (2021). A comparison of microbial communities and volatile compounds in wheat Qu from different geographic locations. LWT.

[27] Kim D., Langmead B., Salzberg S.L. (2015). HISAT: A fast spliced aligner with low memory requirements. Nat. Methods.

[28] Li B., Dewey C.N. (2011). RSEM: Accurate transcript quantification from RNA-Seq data with or without a reference genome. BMC Bioinform..

[29] Love M.I., Huber W., Anders S. (2014). Moderated estimation of fold change and dispersion for RNA-seq data with DESeq2. Genome Biol..

[30] Nissen P., Nielsen D., Arneborg N. (2003). Viable *Saccharomyces cerevisiae* cells at high concentrations cause early growth arrest of non-*Saccharomyces* yeasts in mixed cultures by a cell-cell contact-mediated mechanism. Yeast.

[31] Zhang H., Li J., Xu X., Zhang X., Lan W., Wang Y., Gao X. (2024). Volatile compositions and sensorial properties of strawberry fruit wines fermented with *Torulaspora delbrueckii* and *Saccharomyces cerevisiae* in sequential and simultaneous inoculations. Eur. Food Res. Technol..

[32] San-Juan F., Ferreira V., Cacho J., Escudero A. (2011). Quality and aromatic sensory descriptors (mainly fresh and dry fruit character) of spanish red wines can be predicted from their aroma-active chemical composition. J. Agric. Food Chem..

[33] Hazelwood L.A., Daran J.M., Van Maris Antonius J.A., Pronk Jack T., Dickinson J.R. (2008). The Ehrlich Pathway for Fusel Alcohol Production: A Century of Research on *Saccharomyces cerevisiae* Metabolism. Appl. Environ. Microbiol..

[34] Hirst M.B., Richter C.L. (2016). Review of aroma formation through metabolic pathways of *Saccharomyces Cerevisiae* in beverage fermentations. Am. J. Enol. Vitic..

[35] Eden A., Van Nedervelde L., Drukker M., Benvenisty N., Debourg A. (2001). Involvement of branched-chain amino acid aminotransferases in the production of fusel alcohols during fermentation in yeast. Appl. Microbiol. Biotechnol..

[36] Kłosowski G., Mikulski D., Macko D., Miklaszewska B., Kotarska K., Czupryński B. (2015). Influence of various yeast strains and selected starchy raw materials on production of higher alcohols during the alcoholic fermentation process. Eur. Food Res. Technol..

[37] Liu J., Liu M., Ye P., He C., Liu Y., Zhang S., Cai L. (2022). Ethyl esters enhancement of Jinchuan pear wine studied by coculturing *Saccharomyces bayanus* with *Torulaspora delbrueckii* and their community and interaction characteristics. Food Biosci..

[38] Wang Y.P., Wei X.Q., Guo X.W., Xiao D.G. (2020). Effect of the deletion of genes related to amino acid metabolism on the production of higher alcohols by *Saccharomyces Cerevisiae*. BioMed Res. Int..

[39] Choi Y.J., Lee J., Jang Y.S., Lee S.Y. (2014). Metabolic Engineering of Microorganisms for the Production of Higher Alcohols Metabolic Engineering of Microorganisms for the Production of Higher Alcohols. MBio.

[40] Pires E.J., Teixeira J.A., Brányik T., Vicente A.A. (2014). Yeast: The soul of beer’s aroma-a review of flavour-active esters and higher alcohols produced by the brewing yeast. Appl. Microbiol. Biotechnol..

[41] Giorello F., Valera M.J., Martin V., Parada A., Salzman V., Camesasca L., Dellacassa E. (2019). Genomic and transcriptomic basis of Hanseniaspora vineae’s impact on flavor diversity and wine quality. Appl. Environ. Microbiol..

[42] Jin Z., Wong A., Foo J.L., Ng J., Cao Y.X., Chang M.W., Yuan Y.J. (2016). Engineering *Saccharomyces cerevisiae* to produce odd chain-length fatty alcohols. Biotechnol. Bioeng..

[43] Bornscheuer U.T. (2002). Microbial carboxyl esterases: Classification, properties and application in biocatalysis. FEMS Microbiol. Rev..

[44] Balachandran N., To F., Berti P.J. (2017). Linear Free Energy Relationship Analysis of Transition State Mimicry by 3-Deoxy-d-arabino-heptulosonate-7-phosphate (DAHP) Oxime, a DAHP Synthase Inhibitor and Phosphate Mimic. Biochemistry.

[45] Wang H., Cui Z.F. (2009). Regulation of Shikimic Acid Biosynthesis Pathway. Biotechnol. Bull..

