# Supplementary material for: Dissecting Interactions of Saccharomyces cerevisiae and Pichia kudriavzevii to Shape Kiwifruit Wine Flavor
Source: Foods. 2024 Dec 17;13(24):4077. doi: 10.3390/foods13244077 (PMC11675217; doi:10.3390/foods13244077)
Supplement: Supplementary file 1 [file foods-13-04077-s001.zip › foods-3330053-supplementary.pdf]

Supplementary file

Dissecting Interactions of *Saccharomyces cerevisiae* and *Pichia kudriavzevii* to Shape Kiwifruit Wine Flavor

Yi-Wen Wang [1,2], Yi-Fen Huang [2], Ya-Qi Guo [2], Li Sun [2], Zhi-Lin Jiang [1,2], Yuan-Ting Zhu [2], Rui-Qi Zeng [2], Qi Li [2], Chen Xiao [2,*] and Yong Zuo [1,2,*]

[1] Key Laboratory of the Evaluation and Monitoring of Southwest Land Resources (Ministry of Education), Sichuan Normal University, Chengdu 610101, China; 20221501047@stu.sicnu.edu.cn (Y.-W.W.); jzl13438550280@163.com (Z.-L.J.)

[2] College of Life Science, Sichuan Normal University, Chengdu 610101, China; 20211501042@stu.sicnu.edu.cn (Y.-F.H.); 15883436538@163.com (Y.-Q.G.); sl4104@163.com (L.S.);
zhuyuantingwode@163.com (Y.-T.Z.); 20230054@sicnu.edu.cn (R.-Q.Z.); xiaoqi@sicnu.edu.cn (Q.L.)

* Correspondence: xiaochen_2022@sicnu.edu.cn (C.X.); skzuoyong@sicnu.edu.cn (Y.Z.)

Supplementary Figure S1

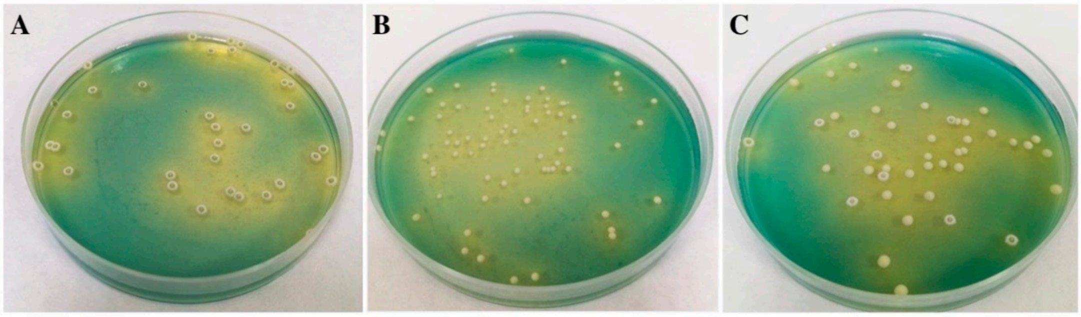

Figure S1 Colony morphologies of different yeast strains on WL identification medium. (A) Group S: single colony morphology of *S. cerevisiae*; (B) Group P: single colony morphology of *P. kudriavzevii*; (C) Group MIX: mixed colony morphology of *S. cerevisiae* and *P. kudriavzevii*.

Supplementary Figure S2

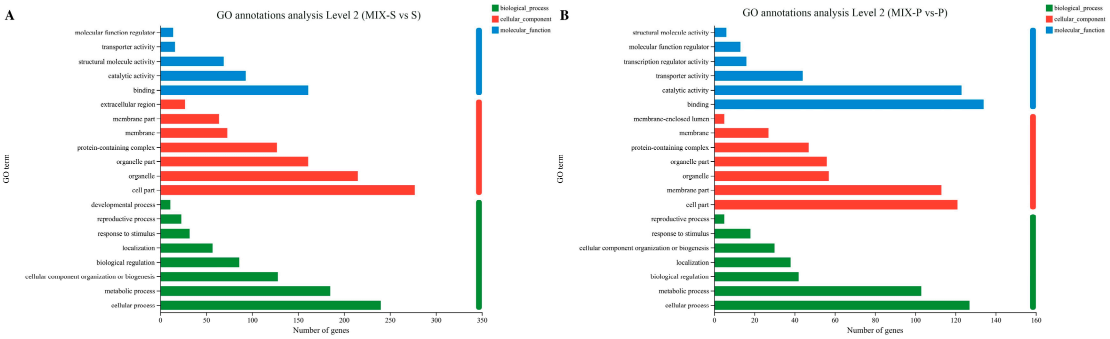

Figure S2 GO analysis of differential genes. (A) group MIX-S vs group S, (B) group MIX-P vs group P.

Supplementary Table S1

Table S1 Genes with significant expression differences between S vs MIX-S

Pathway Definition	Gene id	Gene name	Log2 FC
Glycolysis/Gluconeogenesis	gene-YHR174W	*ENO2*	2.78
	gene-YJR009C	*TDH2*	2.46
	gene-YGR192C	*TDH3*	2.08
	gene-YKL060C	*FBA1*	1.96
	gene-YGR254W	*ENO1*	1.82
	gene-YOL086C	*ADH1*	1.73
	gene-YBR196C	*PGI1*	1.66
	gene-YKL152C	*GPM1*	1.57
	gene-YAL038W	*CDC19*	1.53
	gene-YDR050C	*TPI1*	1.53
	gene-YCR012W	*PGK1*	1.39
	gene-YMR303C	*ADH2*	1.38
	gene-YPL061W	*ALD6*	1.25
Biosynthesis of unsaturated fatty acids	gene-YLR372W	*ELO3*	1.23
	gene-YCR034W	*ELO2*	1.17
Pyruvate metabolism	gene-YOL086C	*ADH1*	1.73
	gene-YAL038W	*CDC19*	1.53
	gene-YMR303C	*ADH2*	1.38
	gene-YPL061W	*ALD6*	1.25
Pentose phosphate pathway	gene-YKL060C	*FBA1*	1.96
	gene-YBR196C	*PGI1*	1.66
Pantothenate and CoA biosynthesis	gene-YPL061W	*ALD6*	1.25
Tyrosine metabolism	gene-YOL086C	*ADH1*	1.73
	gene-YMR303C	*ADH2*	1.38
Lysine degradation	gene-YNR050C	*LYS9*	1.67
	gene-YPL061W	*ALD6*	1.25
Lysine biosynthesis	gene-YNR050C	*LYS9*	1.67
Arginine and proline metabolism	gene-YPL061W	*ALD6*	1.25
	gene-YLR142W	*PUT1*	1.14

Histidine metabolism	gene-YPL061W	*ALD6*	1.25
Valine，leucine and isoleucine degradation	gene-YPL061W	*ALD6*	1.25
Phenylalanine metabolism	gene-YEL066W	*HPA3*	1
Tryptophan metabolism	gene-YPL061W	*ALD6*	1.25
Glycine，serine and threonine metabolism	gene-YKL152C	*GPM1*	1.57
Citrate cycle (TCA cycle)	gene-YJL045W	*SDH9*	-1.25
Glyoxylate and dicarboxylate metabolism	gene-YDR019C	*GCV1*	-1.09
	gene-YMR189W	*GCV2*	-1.43
	gene-YLR058C	*SHM2*	-1.46
Glycine，serine and threonine metabolism	gene-YDR019C	*GCV1*	-1.09
	gene-YER081W	*SER3*	-1.32
	gene-YMR189W	*GCV2*	-1.43
	gene-YLR058C	*SHM2*	-1.46
Alanine，aspartate and glutamate metabolism	gene-YMR300C	*ADE4*	-1.15
	gene-YLR359W	*ADE13*	-1.22
Cysteine and methionine metabolism	gene-YGL184C	*STR3*	-1.07
	gene-YER081W	*SER3*	-1.32
Arginine biosynthesis	gene-YJL088W	*ARG3*	-1.11

Supplementary Table S2

Table S2 Genes with significant expression differences between MIX-P vs P

Pathway Definition	Gene id	Gene name	Log2 FC
Glycolysis/Gluconeogenesis	gene-C5L36_0D04320	*ADH*	4.03
	gene-C5L36_0D04330	*ADH*	3.95
	gene-C5L36_0A10330	*PDC，ARO10*	2.93
	gene-C5L36_0B06500	*PGM*	2
	gene-C5L36_0C11530	*PDC，ARO10*	1.86
	gene-C5L36_0C10060	*adhP*	1.83
	gene-C5L36_0E01970	*pfkA，PFK*	1.79
	gene-C5L36_0A12480	*PGK，pgk*	1.17
	gene-C5L36_0C08610	*adhP*	1.09
	gene-C5L36_0B12280	*AKR1A1，ADH*	1
Biosynthesis of unsaturated fatty acids	gene-C5L36_0A08380	*ACAA1*	1.68
Pyruvate metabolism Pyruvate metabolism	gene-C5L36_0D04320	*AKR1A1，ADH*	4.03
	gene-C5L36_0D04330	*AKR1A1，ADH*	3.95
	gene-C5L36_0D04970	*hchA*	3.83
	gene-C5L36_0A09950	*ACH1*	2.48
	gene-C5L36_0C10060	*adhP*	1.83
	gene-C5L36_0E04340	*MDH2*	1.43
	gene-C5L36_0A12710	*MDH2*	1.23
	gene-C5L36_0C08610	*adhP*	1.09
	gene-C5L36_0B12280	*AKR1A1，adh*	1
Pentose phosphate pathway	gene-C5L36_0B06500	*PGM*	2
	gene-C5L36_0E01970	*pfkA，PFK*	1.79
	gene-C5L36_0B01030	*talA，talB*	1.01
Citrate cycle （TCA cycle）	gene-C5L36_0A01330	*CS，gltA*	2.6
	gene-C5L36_0B11140	*LSC1*	1.62
	gene-C5L36_0E04340	*MDH2*	1.43
	gene-C5L36_0C08340	*ACO，acnA*	1.38
	gene-C5L36_0C04360	*IDH3*	1.31
	gene-C5L36_0A12710	*MDH2*	1.23
Glyoxylate and dicarboxylate metabolism	gene-C5L36_0A01330	*CS，gltA*	2.6
	gene-C5L36_0B10830	*aceA*	1.95
	gene-C5L36_0E04340	*MDH2*	1.43
	gene-C5L36_0C08340	*ACO，acnA*	1.38
	gene-C5L36_0A12710	*MDH2*	1.23
Ascorbate and aldarate metabolism	gene-C5L36_0D04320	*AKR1A1，ADH*	4.03
	gene-C5L36_0D04330	*AKR1A1，ADH*	3.95
	gene-C5L36_0B01460	*MIOX*	1.4

	gene-C5L36_0B12280	*AKR1A1，ADH*	1
Pentose and glucuronate interconversions	gene-C5L36_0D04320	*AKR1A1，ADH*	4.03
	gene-C5L36_0D04330	*AKR1A1，ADH*	3.95
	gene-C5L36_0D03580	*ARD*	2.81
	gene-C5L36_0B08750	*UGP2，galU，galF*	1.46
	gene-C5L36_0B11990	*XR*	1.18
	gene-C5L36_0B12280	*AKR1A1，ADH*	1
Arginine and proline metabolism	gene-C5L36_0C04660	*DAO，aao*	1.18
Valine，leucine and isoleucine degradation	gene-C5L36_0A08380	*ACAA1*	1.68
Tryptophan metabolism	gene-C5L36_0E01100	*ARO8*	1.24
Cysteine and methionine metabolism	gene-C5L36_0E04340	*MDH2*	1.43
	gene-C5L36_0E01100	*ARO8*	1.24
	gene-C5L36_0A12710	*MDH2*	1.23
	gene-C5L36_0A06070	*metK*	1.09
	gene-C5L36_0B00970	*cysK*	1.04
Fatty acid biosynthesis	gene-C5L36_0C10060	*adhP*	1.83
Tyrosine metabolism	gene-C5L36_0C10060	*adhP*	1.83
	gene-C5L36_0A08590	*HPD，hppD*	1.42
	gene-C5L36_0E01100	*ARO8*	1.24
	gene-C5L36_0C08610	*adhP*	1.09
Lysine biosynthesis	gene-C5L36_0E01100	*ARO8*	1.24
Arginine biosynthesis	gene-C5L36_0E00260	*OTC，argF，argI*	3.6
	gene-C5L36_0D02650	*gdhA*	1.92
	gene-C5L36_0C10830	*ARG2*	1.35
	gene-C5L36_0B12110	*argG，ASS1*	1.11
	gene-C5L36_0A07860	*argH，ASL*	1.07
Phenylalanine metabolism	gene-C5L36_0A10330	*PDC，ARO10*	2.93
	gene-C5L36_0C11530	*PDC，ARO10*	1.86
	gene-C5L36_0A08590	*HPD，hppD*	1.42
	gene-C5L36_0E01100	*ARO8*	1.24
Phenylalanine，tyrosine and tryptophan biosynthesis	gene-C5L36_0E01100	*ARO8*	1.24
Glycine，serine and threonine metabolism	gene-C5L36_0C04660	*DAO，aao*	1.18
Alanineaspartate and glutamate metabolism	gene-C5L36_0D02650	*E1.4.1.4，gdhA*	1.92
	gene-C5L36_0B12110	*argG，ASS1*	1.11
	gene-C5L36_0A07860	*argH，ASL*	1.07
Fatty acid biosynthesis	gene-C5L36_0A07460	*fabF，OXSM，CEM1*	-1.52
Pantothenate and CoA biosynthesis	gene-C5L36_0A04800	*ilvD*	-1.15

Tyrosine metabolism	gene-C5L36_0A05370	*AOC3，AOC2，tynA*	-1.94
Lysine biosynthesis	gene-C5L36_0A01390	*ACO2*	-1.16
Arginine biosynthesis	gene-C5L36_0A03160	*GPT，ALT*	-1.2
Valine，leucine and isoleucine biosynthesis	gene-C5L36_0A04800	*ilvD*	-1.15
Phenylalanine metabolism	gene-C5L36_0A05370	*AOC3，AOC2，tynA*	-1.94
Phenylalanine，tyrosine and tryptophan biosynthesis	gene-C5L36_0A01580	*aroC*	-1.56
Glycine，serine and threonine metabolism	gene-C5L36_0A05790<br>gene-C5L36_0A05370	*ydfG*<br>*AOC3，AOC2，tynA*	-1.88<br>-1.94
Alanineaspartate and glutamate metabolism	gene-C5L36_0A03160<br>gene-C5L36_0A02440	*GPT，ALT*<br>*purB，ADSL*	-1.2<br>-2.01
beta-Alanine metabolism	gene-C5L36_0A05370	*AOC3，AOC2，tynA*	-1.94

Supplementary Table S3

Table S3 The volatile compounds of different fermentation groups (MIX, S, P)

Compounds (μg/L)	MIX	S	P	Foremost 20 differentiated volatile compounds
Isoamyl alcohol	1018.75±45.44	1263.34±85.94	737.37±52.93	*
2-Octanol	822±0	822±0	822±0	
Phenylethyl alcohol	687.88±24.42	753.27±43.13	825.92±54.63	*
Octanoic acid	981.1±106.34	963.97±86.4	139.81±24.17	*
Ethyl acetate	95.33±67.45	nd	1497.08±844.03	*
2,4-Di-tert-butylphenol	294.89±20.62	375.2±15.36	221.63±21.69	*
Decanoic acid	333.88±115.93	337.09±139.44	26.56±1.93	*
Ethyl carprylate	260.73±23.69	228.5±35.9	13.01±8	*
3,4-Dimethylbenzaldehyde	203.61±44.56	207.8±48.42	80.97±14.95	*
Phenylethyl acetate	148.75±8.85	134.51±14.3	81.88±40.36	*
Isobutanol	70.7±6.95	93.66±9.26	130.26±20.18	*
Hexanoic acid	145.33±10.33	129.46±14.13	14.97±11.08	*
Ethyl caprate	135.38±25.7	150.56±33.69	2.81±3.98	*
Alpha-Terpineol	79.7±4.26	80.32±8.13	69.24±12.93	
Diethyl Phthalate	81.98±2.52	78.38±4.11	62.91±4.09	
Isoamyl acetate	70.35±5.7	62.48±8.76	20.63±9.43	*
Linalool	73.85±104.44	66.22±93.65	nd	
Ethyl 3-phenylpropionate	56.25±4.5	49.9±10.3	9.99±14.13	*
Nonanal	38.85±3.21	38.07±1.27	28.82±5.4	
Nonanoic acid	35.17±0.41	26.98±5.01	28.1±3.63	*

Ethyl hexanoate	40.56±1.2	36.05±3.12	10.4±1.78	
1-Octanol	26.41±1.79	29.97±7.89	14.54±1.45	
2-Methoxy-4-vinylphenol	29±3.44	32.21±3.88	nd	
2-Octanone	18.54±0.27	17.06±1.83	24.72±2.02	
Isobutyric acid	10.52±7.52	26.72±13.2	21.15±4.64	*
Methyl octanoate	25.86±1.43	21.25±2.47	nd	
Methyl caprate	19.71±2.62	19.05±3.44	0.43±0.61	
Ethyl 9-decenoate	19.16±3.35	14.59±10.65	nd	
2,6-Di-tert-butyl-4-hydroxy-4-methylcyclohexa-2,5-dien-1-one	11.83±0.6	11.39±0.58	10.11±1.63	
2-Ethyl-1-hexanol	10.56±0.45	10.22±0.32	12.44±0.53	
Ethyl butyrate	18.6±1.65	nd	13.67±10.01	*
Acetic acid	11.97±2.08	12.19±2.51	7.26±1.22	
2-Methyl-propanoic acid 3-hydroxy-2,2,4-trimethylpentyl ester	10.66±0.85	10.25±0.22	10.39±1	
2,6,6-Trimethyl-1,3-cyclohexadiene-1-carboxaldehyde	9.84±0.6	10.85±0.99	9.47±0.24	
n-Tridecanol	7.4±0.68	9.38±0.32	6.11±1.12	
Ethyl hexadecanoate	6.82±2.47	7.2±0.67	6.77±1.55	
3-Methyl-5-propylnonane	15.32±0.47	5.22±7.39	nd	*
Hexanol	7.09±0.45	6.2±0.51	6.77±1.7	
2-Ethyl-hexanoic acid	7.81±1	6.27±1.2	5.46±0.61	
Pentadecane	nd	9.88±6.99	9.36±1	*
2,2,4-Trimethyl-1,3-pentanediol diisobutyrate	6.52±0.46	6.58±0.43	4.73±1.51	
Benzeneacetaldehyde	3.93±0.52	4.76±0.86	8.56±0.64	
Ethyl benzoate	7.04±0.52	7.1±0.18	3±1.06	
2-Nonanol	8.77±0.22	5.65±0.54	nd	
Heptadecane	5.81±2.97	6.14±2.31	2.04±1.47	
Heneicosane	3.5±2.51	1.72±2.43	7.54±4.49	

Diisobutyl phthalate	4.47±0.07	4.3±0.08	3.6±0.18	
Dodecane	4.86±3.46	3.22±2.99	3.77±2.67	
Isoamyl octanoate	4.93±6.98	5.8±8.2	nd	
Benzoic acid	6.64±4.84	nd	3.45±0.22	*
Ethyl phenylacetate	nd	nd	9.36±1.51	
4-Isopropoxy-2-butanone	2.37±0.24	2.6±0.89	3.46±0.89	
Methyl hexadecanoate	2.23±0.83	2.2±0.11	3.86±1.05	
1-Decanol	3.23±0.05	3.86±0.31	nd	
3-Octanol	nd	nd	6.03±1.85	
Ethyl trans-4-decenoate	nd	5.58±7.89	nd	
3,5-di-tert-Butyl-4-hydroxybenzaldehyde	1.67±0.04	2±0.27	1±0.7	
2-Undecanone	1.47±2.07	2.83±2.02	nd	
Ethyl linoleate	1.56±1.14	1.5±1.07	0.85±1.2	
Benzaldehyde	nd	nd	3.47±4.9	
Octadecane	1.83±2.59	1.61±2.27	nd	
D-Limonene	nd	1.62±2.3	1.77±2.5	
Ethyl lactate	nd	nd	3.13±0.35	
Toluene	0.93±1.31	nd	1.94±1.52	
2,5-Dimethyl-dodecane	2.85±4.03	nd	nd	
3-Ethoxy-1-propanol	2.81±3.97	nd	nd	
β-Damascenone	1.92±1.36	0.86±1.22	nd	
2,5-Dimethyl-benzaldehyde	nd	2.68±3.79	nd	
Octanal	1.25±1.77	1.18±1.67	nd	
gamma-Decalactone	nd	1.42±1.05	0.76±1.08	
cis-3,3,5-trimethylcyclohexyl acetate	nd	nd	2.1±1.52	
Methyl benzoate	nd	nd	1.76±2.49	

trans-Geranylgeraniol	nd	nd	1.56±1.1
4-Methyl-1-pentanol	nd	1.48±0.17	nd
Tetradecane	nd	1.4±1.98	nd
Hexadecane	nd	nd	1.46±1.18
Ethyl oleate	nd	nd	0.92±1.3
(E)-Hexadec-2-enal	nd	0.9±1.28	nd
4-Propylphenol	nd	nd	0.79±1.12
Geranic acid	0.75±1.06	nd	nd
2-Methyldodecane	nd	nd	0.74±1.04
5-Methylundecane	nd	0.69±0.97	nd
(E)-9-Octadecenoic acid ethyl ester	nd	nd	0.5±0.71
Benzothiazole	nd	nd	0.4±0.56

Note: “*” represents foremost 20 differentiated volatile compounds (based on their VIP values) were selected by PLS-DA model (simca 14.1); “nd” represents that the compounds had not been detected.
